# Interleukin‐6 signaling requires only few IL‐6 molecules: Relation to physiological concentrations of extracellular IL‐6

**DOI:** 10.1002/iid3.292

**Published:** 2020-02-27

**Authors:** Morten B. Hansen

**Affiliations:** ^1^ Department of Clinical Immunology Rigshospitalet, Copenhagen University Hospital Copenhagen Denmark

**Keywords:** bioactivity, cytokines, IL‐6, interleukins, receptor occupancy

## Abstract

**Introduction:**

The aim of this study was to give quantitative insight into the number of cytokine molecules needed to activate a target cell and relate this to the physiological consequences of the amounts of cytokines typically detectable in humans. As a model system blood interleukin‐6 (IL‐6) was chosen since this cytokine is one of the most studied and clinically monitored cytokines, and because of the tools for the present investigations such as fully bioactive iodinated recombinant IL‐6, cellular cytokine binding assays, and bioassays have been thoroughly validated.

**Methods:**

The key intermediates of the basic equilibrium principles that govern cytokine binding and exchange were deduced and applied on concrete estimations of cellular and extracellular IL‐6 binding in the bloodstream based on experimental binding data and data from the literature. In parallel, in vitro cellular IL‐6 binding data was substantiated by paired measurements of IL‐6 bioactivity on IL‐6 sensitive B9 hybridoma cells.

**Results:**

Blood leucocytes and B9 cells expressed 50 to 300, 10 to 20 picomolar affinity, IL‐6 binding sites per cell and at physiological concentrations of IL‐6 less than 10 IL‐6 molecules seemed to be bound to blood cells. Nonetheless, binding off as few as four IL‐6 molecules per cell seemed to result in statistically significant bioactivity, whereas binding of 16 IL‐6 molecules triggered extensive cellular responses.

**Conclusion:**

Together, the estimations and the measurements support the notion that target cells with more than 100 bioactive cytokine receptors per cell, such as T cells and hepatocytes, are likely to be under steady and substantial cytokine‐induced endocrine activation.

## Graphical abstract

The paper gives quantitative insight into the number of cytokine molecules needed to activate a target cell, and relate this to the physiological consequences of the amounts of cytokines typically detectable in humans.

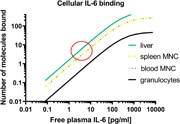

**Abbreviation**
rhIL‐6: recombinant human interleukin‐6

## 1 INTRODUCTION

The number of cytokine molecules that have to bind to target cell receptors to elicit cellular responses and the biological significance in this context of different plasma concentrations of cytokines is largely unknown. On the basis of various theoretical plasma concentrations of the cytokine interleukin‐6 (IL‐6), the present study enlightened these topics.

IL‐6 is produced by a wide variety of cells and acts via receptors on target cells, for example, the liver, blood, and immune system. The cellular IL‐6 receptor system consists of the ligand‐binding chain (CD126) and the signal‐transducing chain (CD130).^1^ The cellular signaling complex is a hexamer, formed by the association of two IL‐6‐CD126‐CD130 trimers. CD130 can transduce both *cis*‐ and *trans*‐signaling via membrane‐bound and soluble forms of CD126, respectively.^2^, ^3^, ^4^, ^5^, ^6^, ^7^, ^8^, ^9^ In this study, all the blood's IL‐6 binding factors, cellular as well as extracellular, were assumed to competitively bind to the same regions of the IL‐6 molecule and cell membrane receptors were assumed to be uniformly distributed on the cell surface.^10^, ^11^ The formulas deduced were based on fundamental chemical equilibrium rules described by Feldman^12^ and Munson and Rodbard.^13^, ^14^

High‐affinity cytokine binding is characterized by relatively fast association kinetics and correspondingly slow dissociation kinetics and equilibrium typically occurs within seconds to minutes.^15^, ^16^ IL‐6 molecules present in the bloodstream are reversibly exchanged between potential binding factors and exist as either bound or free molecules. The overall IL‐6 mediated bioactivity in such a well‐defined and well‐mixed compartment is hence depending on (a) the available extracellular concentration of IL‐6 and (b) the binding activity and consequently the bioactivity of cellular and extracellular IL‐6 binding factors.

The binding activity of various binding factors must be considered by basic equilibrium principles: when cytokine molecules (*CK*) bind reversibly to binding factor molecules (*R*) complexes (*CKR*) are formed:

CK+R⟷CKR

The concentration of bound cytokine, [*CKR*], depends on the concentrations of free *CK* and the concentration of *R* as well as the binding affinity. At equilibrium, the dissociation constant, *Kd*, defines the binding affinity:

$$\mathrm{Kd}=\frac{\left[\mathrm{CK}\right]\left[R\right]}{\left[\mathrm{CKR}\right]}$$

Here, [*CK*] is the activity of the free, unbound, cytokine, with the molar concentration *F*. [*CKR*] is the molar concentration of the bound cytokine (*B*). [*R*] is the molar concentration of the available binding capacity of the cytokine binding factor which again is the maximum binding capacity of the binding factor (*B*_max_), minus the concentration of bound cytokine (*B*). Accordingly, [*R*] has the molar concentration *B*_max_ *− B*.

$$\mathrm{Kd}=\frac{\left[\mathrm{CK}\right]\left[R\right]}{\left[\mathrm{CKR}\right]}\approx \frac{F\left(B_{\max}-B\right)}{B}⟺$$

Notice that *Kd* on the left side of the equal sign is a constant and that all parts on the right side of the equal sign are variables. Therefore, changing one of these variables will affect the other variables, since *Kd* must be constant.

The concentration of the cytokine binding factor that is in complex with the cytokine (*B*) can be expressed relative to the total binding capacity of the cytokine binding factor (*B*_max_). This ratio is the degree of saturation (*B*/*B*_max_) of the cytokine binding factor, and it is an important key intermediate score, which is used to estimate the amount of bound and hence potential bioactive cytokine. *B*/*B*_max_ can be deduced from the above, dividing by *F* and *Kd* and multiplying by *B* on both sides of the equal sign:

$$\frac{B}{F}=\frac{B_{\max}-B}{\mathrm{Kd}}⟺$$

Then divided by *B*_max_ and multiplied by *F* on both sides of the equal sign:

$$\frac{\frac{B}{B_{\max}}}{F}=\frac{1-\left(\frac{B}{B_{\max}}\right)}{\mathrm{Kd}}⟺$$

$$\frac{B}{B_{\max}}=\frac{F-F\left(\frac{B}{B_{\max}}\right)}{\mathrm{Kd}}⟺$$

$$\left(\frac{B}{B_{\max}}\right)\mathrm{Kd}=F-F\left(\frac{B}{B_{\max}}\right)⟺$$

$$\left(\frac{B}{B_{\max}}\right)\left(\mathrm{Kd}+F\right)=F⟺$$

$$\frac{B}{B_{\max}}=\frac{F}{\mathrm{Kd}+F}\left(t\text{he degree of saturation}\right)$$

Notice how *B*/*B*_max_ solely depends on (a) *F*, the free (measurable) concentration of the cytokine and (b) *Kd*, the affinity of the cytokine binding factor. Thus, at equilibrium, the degree of saturation (*B*/*B*_max_) of a putative binding factor can be calculated when one has measured the concentration of free cytokine (*F*) and knows the *Kd* of the binding factor in question. In this context, *F* could be a certain plasma concentration of IL‐6, and *Kd* could be the affinity of IL‐6 receptors on polymorph nuclear granulocytes (PNGs). The estimate of the degree of saturation (calculated as *F*/(*Kd* + *F*)) is the key intermediate result in this context since this estimate can be converted directly to an estimate of the absolute amount of cytokine bound to the binding factor in question (*B*) when knowing the concentration of this binding factor (*B*_max_):

$$B=\;The\,\;\text{deg}ree\,\;of\,\;saturation\;\cdot \;B_{\max}=\left(\frac{F}{\mathrm{Kd}+F}\right)\;\cdot \;B_{\max}.$$

In the bloodstream, multiple diverse cytokine binding factors are present simultaneously, such as various cytokine receptor‐bearing cells and soluble receptors, and at equilibrium, the total cytokine binding (*B*_total_) is distributed among these binding factors. Therefore, the total cytokine binding (*B*_total_) is equal to the sum of the individual binding factors cytokine binding:

$$B_{total}=B_{1}+B_{2}+B_{3}+\text{⋯}\;+B_{n}\Leftrightarrow \;$$

$$\frac{B_{total}}{F}=\frac{B_{1}}{F}+\frac{B_{2}}{F}+\frac{B_{3}}{F}+\text{⋯}\;+\frac{B_{n}}{F}\Leftrightarrow \;$$

$$\frac{B_{total}}{F}=\frac{B_{\max1}}{{\mathrm{Kd}}_{1}+F}+\frac{B_{\max2}}{{\mathrm{Kd}}_{2}+F}+\frac{B_{\max3}}{{\mathrm{Kd}}_{3}+F}+\text{⋯}\;+\frac{B_{\max n}}{{\mathrm{Kd}}_{n}+F}$$

In the bloodstream, *B*_1_ could be the IL‐6 binding to PNGs, *B*_2_ could be the IL‐6 binding to mononuclear cells (MNCs), *B*_3_ could be the IL‐6 binding to soluble receptors, and so on. Note that the measured concentration of the free cytokine (*F*) has the same numerical value on both sides of the equal sign independent of how many distinct binding factors that are present or considered. The above equation expresses that the individual binding factors, at equilibrium and hence, at a constant value of *F*, have bound their respective proportion of the total amount of cytokine in the compartment, and that these proportions can be estimated when knowing the binding capacities of the binding factors (*B*_max_) and their respective affinities for the cytokine (*Kd*).

Conclusion: Having measured a concentration of free cytokine (*F*) in for instance plasma, the corresponding cytokine binding (*B′*) to a putative cytokine binding factor present in the blood can be estimated when knowing the concentration (*B*_max′_) and the affinity (*Kd′*) of this binding factor. First, the degree of saturation (*B′*/*B*_max′_) of the putative binding factor is calculated (as *F*/(*Kd’* + *F*)). Second, the concentration of bound cytokine (*B′*) to this binding factor is calculated (as *B’* = degree of saturation × *B*_max′_).

## 2 MATERIALS AND METHODS

### 2.1 Isolation of blood MNCs and PNGs

Blood samples were obtained from healthy blood donors at Rigshospitalet, Copenhagen, Denmark after informed consent and collected in vacutainer tubes (BD Bioscience, San Jose, CA), containing ethylenediaminetetraacetic acid as an anticoagulant. PBMC and PNG were isolated from healthy O rhesus D‐positive blood donors by LeucoSep and Ficoll‐Paque Premium density‐gradient centrifugation (Greiner Bio‐One). Blood MNCs were suspended in Roswell Park Memorial Institute (RPMI) 1640 medium containing 25 mM HEPES (Gibco) and supplemented with 0.8 mM l‐glutamine and 2.5 mg/mL gentamicin (Sigma‐Aldrich, Denmark). Cells were utilized in binding experiments immediately after the isolation procedure.

### 2.2 The mouse B cell hybridoma cell line B9

B9 cells (European Collection of Cell Cultures, Wiltshire, UK) were propagated in RPMI 1640 medium supplemented with 2 mM l‐glutamine (Sigma‐Aldrich, Darmstadt, Germany), 50 µM 2‐mercaptoethanol (Sigma‐Aldrich), 5% heat‐inactivated fetal calf serum (Invitrogen), 400 IU/mL penicillin/400 µg/mL streptomycin (Sigma‐Aldrich), and 5 pM recombinant human interleukin‐6 (rhIL‐6; PeproTech, Stockholm, Sweden) at 37°C in 5% CO_2_‐humidified air. Before use, the cells were washed five times by repeated centrifugation and resuspended in 10 mL of IL‐6 free medium.^17^

### 2.3 3‐(4,5‐Dimethylthiazol‐2‐yl)‐2,5‐diphenyltetrazolium bromide bioassay

Assays were performed using flat‐bottomed microtiter plates (Nunc, Roskilde, Denmark). The dehydrogenase activity of B9 cells incubated with labeled and unlabeled rhIL‐6 was determined by the 3‐(4,5‐dimethylthiazol‐2‐yl)‐2,5‐diphenyltetrazolium bromide (MTT) assay. In brief, 5000 B9 cells/well were incubated with serial dilutions of sterile‐filtered labeled or unlabeled rhIL‐6 in a total volume of 110 µL for 72 hours (37°C, 5% CO_2_), after which the MTT assay was performed as described previously.^18^

### 2.4 Iodination of IL‐6

Carrier‐free, *Escherichia coli*‐derived rhIL‐6 (PeproTech) was labeled with ^125^I using the chloramine‐T method and subsequently purified by molecular‐size chromatography using a Sephadex® G‐75 Superfine column (GE Healthcare, Brøndbyvester, Denmark). In brief, 5 µL of rhIL‐6 (5 × 10^−6^M), 5 µL of 0.2M NaH_2_PO_4_, pH 6.9 (Merck, Whitehouse Station, NJ), 2 µL of Na^125^I (3.5 Gbq/mL; GE Healthcare, Amersham, UK) and 5 µL of 12.8 mg/mL chloramine‐T (Merck) were incubated for 20 minutes (20°C), after which 183 µL of 1% BSA (Sigma‐Aldrich) and 0.1% PEG6000 (Merck) in H_2_O were added.^19^ The ^125^I‐rhIL‐6 was more than 95% monomeric, and the specific activity varied between 1.5 × 10^18^ and 2.1 × 10^18^ cpm/mol. The molecular weight of rhIL‐6 was 22 000 g/mol.

### 2.5 Cell binding assay

Cells were washed twice in fresh medium containing 1% human serum albumin (HSA; Behringwerke, Marburg, Germany). To reduce the amount of any bound rhlL‐6 from the IL‐6R, the cells were resuspended in 10 mM sodium citrate in 0.9% NaCl, pH 4.0, agitated for 25 seconds and washed twice in medium supplemented with 1% HSA. The binding of rhIL‐6 to cells was assessed by incubating the cells in a final volume of 225 µL culture medium supplemented with varying concentrations of ^125^I‐rhIL‐6 with or without a 1000‐molar excess of unlabeled rhIL‐6 to assess specific cellular tracer binding. After 22 hours at 4°C, 200 µL of cell suspension was applied to bis(‐2‐ethylhexyl)phthalate/dibutyl phthalate 1:1 (Merck) and centrifuged (4°C, 10 000*g*, 10 minutes). Cell‐bound and free tracer molecules were separated by cutting through the oil layer, and the activity of ^125^I was quantified by a 1470 Wizard gamma counter (Wallac, Turku, Finland) with an error of less than 5%.^16^

### 2.6 Further calculations and statistical analyses

Each experiment was repeated three to six times with comparable results. Statistical differences were evaluated by Wilcoxon's test. Cellular tracer binding data were fitted by interpolation and linear regression using the GraphPad Prism software, version 7. The degree of saturation of cellular IL‐6 binding was estimated using the following formula^16^, ^20^:

$$B=\frac{-\;\left(\text{Total}\;+\;B_{\max}+\mathrm{Kd}\right)+\sqrt{{\left(\text{Total}\;+\;B_{\max}+\mathrm{Kd}\right)}^{2}-4\left(\text{Total}\;\times \;B_{\max}\right)}}{2},$$

where “*B*” denotes cell‐bound IL‐6, “Total” is the total IL‐6 concentration (*B* + *F*), “*B*_max_” is the maximal cellular IL‐6 binding capacity, and “*Kd*” is the binding affinity. *B*/*B*_max_ is the degree of saturation of the cellular binding (or the proportion of available binding sites that have bound IL‐6 molecules). For these calculations, Microsoft Excel was used with four significant digits after the comma. The probabilities of different numbers of IL‐6 molecules bound per cell under the given experimental conditions were further qualified statistically by means of the Poisson distribution.

### 2.7 Ethics

Oral and written informed consent was obtained for participating blood donors. This project was approved by both the Danish health research ethics committee system and the Danish Data Protection Agency. The study was in compliance with regional and national guidelines.

### 2.8 Availability of data

The data that support the findings of this study are available from the corresponding author upon reasonable request.

## 3 RESULTS

Below are specific examples regarding IL‐6 binding in the bloodstream based on typical plasma concentrations of free IL‐6. An estimation of the IL‐6 binding is exemplified on MNCs, polymorphonuclear granulocytes (PNG), hepatocytes, and soluble IL‐6 receptors (sIL‐6R).

**Figure 1:**
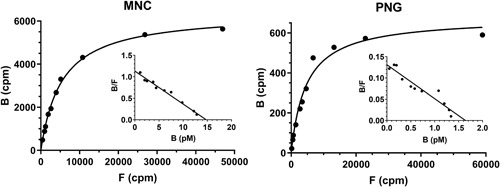
^125^I‐rhIL‐6 binding to mononuclear cells (MNC) and polymorph nuclear granulocytes (PNG). A total of 6.9 × 10^6^ MNC and 4.5 × 10^6^ PNG in 225 µL of media were incubated with varying concentrations of ^125^I‐rhIL‐6 for 22 hours at 4°C, after which specific cell‐bound and free ^125^I‐rhIL‐6 were measured. Insets: MNC bound ^125^I‐rhIL‐6 with a *Kd* ≈ 14 pM and a *B*_max_ ≈ 14.7 pM, corresponding to 289 binding sites per cell (*R* ^2^ = .95; *P* < .001). PNG bound ^125^I‐rhIL‐6 with a *Kd* ≈ 12 pM and a *B*_max_ ≈ 1.6 pM, corresponding to 50 binding sites per cell (*R* ^2^ = .91; *P* < .001). Comparable data were obtained with cells from four individual blood donors, see Table 1

In this context, the number (*B*_max_) and the affinity (*Kd*) of IL‐6 receptors on MNC and PNG were estimated from Scatchard plots (Figure 1 and Table 1a, 1b). *B*_max_ and *Kd* values for hepatocytes and sIL‐6R were obtained from the literature (Table 2).

Example 1 (Mononuclear cells, MNC).

A plasma concentration of free IL‐6 ≈ 2.0 pg/mL has been measured.

*F* = 2 pg/mL ≈ 0.08 pM. The number of IL‐6 binding sites on MNC (*B*_max_) is 284 per cell and the affinity (*Kd*) is 15 pM (Table 1a).

**Table 1a:** IL‐6 binding sites on mononuclear cells

Donor ID	*Kd*		Number of binding sites	
	High avidity	Low avidity	High avidity	Low avidity
1	16 pM	n.d.	275	n.d.
2	12 pM	410 pM	319	250
3	14 pM	n.d.	289	n.d.
4	15 pM	530 pM	251	400

At equilibrium, the degree of saturation of IL‐6 receptors on MNC is:

$$\frac{B}{B_{\max}}=\frac{F}{\mathrm{Kd}+F}=\frac{0.08\;pM}{15\;\text{pM}+0.08\;\text{pM}}=0.5\%.$$

This indicates that 0.5% × 284 binding sites per cell are occupied by IL‐6 or on average, 1.5 cytokine molecules bound per MNC.

The cumulative *B*_max_ from the entire amount of MNC in the bloodstream (equivalent to 6 × 10^9^ MNC per liter of plasma) is:

6 × 10^9^ MNC/l × 284 receptors pr. MNC/6.023 × 10^23^ receptors/mol = 2.83 pM.

Taken together, at a plasma concentration of 2.0 pg/mL (≈0.08 pM), 0.5% × 2.83 pM = 0.015 pM IL‐6 is bound to MNC in the bloodstream. Consequently, at this plasma concentration of free IL‐6, there is more than five times as much free IL‐6 as IL‐6 bound to MNC in the circulation.

Example 2 (polymorphonuclear granulocytes, PNG).

Again *F* = 2.0 pg/mL IL‐6 (≈0.08 pM IL‐6). The number of IL‐6 binding sites on PNG (*B*_max_) is 48 per cell and the affinity (*Kd*) is 15 pM (Figure 1 and Table 1b).

**Table 1b:** IL‐6 binding sites on polymorph nuclear granulocytes

Donor ID	*Kd*		Number of binding sites	
	High avidity	Low avidity	High avidity	Low avidity
1	16 pM	580 pM	63	140
2	12 pM	574 pM	50	340
3	12 pM	308 pM	24	329
4	20 pM	556 pM	57	217

Since IL‐6 receptors on PNG are assumed to be identical to IL‐6 receptors on MNC (with similar *Kd*), the degree of saturation of these receptors is equal to the degree of saturation of IL‐6 receptors on MNC (*B*/*B*_max_ = 0.5%).

The number of IL‐6 molecules bound per PNG ≈ 0.5% × 48 binding sites/PNG = 0.25. For the bloodstream's entire granulocytes, the cumulative PNG IL‐6 binding, *B* ≈ 10 × 10^9^ PNG/l × 48 binding sites/PNG/6.023 × 10^23^ receptors/mol × 0.5% = 0.004 pM and hence approximately four times less than the amount of IL‐6 bound to MNC.

Example 3 (Hepatocytes, the liver)

The corresponding estimate of IL‐6 binding to hepatocytes at a plasma concentration of 2.0 pg/mL is 3.18 molecules bound per cell and 0.37 pM bound to all hepatocytes. It corresponds to more than nine times as much liver bound IL‐6 as free plasma IL‐6. Notice that this binding activity was estimated for unstimulated hepatocytes having a relatively low number of IL‐6 receptors. In this context, 600 IL‐6 binding sites per cell were used (Table 2).

**Table 2:** *B*_max_ and *Kd* for IL‐6 binding factors in the blood

IL‐6 binding factors	*Kd*, pM	Receptor number per cell	*B*_max_ in 3l of plasma, pM
Mononuclear cells	15	284	2.83
Granulocytes	15	48	0.77
Hepatocytes	15	600‐9000	69‐1000
Spleen cells	15	284	4.1
sIL‐6R	5000‐50 000		650‐2000^a^

*Note*: Mononuclear cells, (see Table 1a), blood concentration ≈ 3 × 10^9^ per liter blood ≈ 6 × 10^9^ per liter plasma,^21^; granulocytes, (see Table 1b), blood concentration ≈  5 × 10^9^ per liter blood ≈ 10 × 10^9^ per liter plasma.^21^ Hepatocytes, number in the liver: 139 × 10^9^ per gram liver, liver mass ≈ 1.500 g.^21^, ^22^, ^23^ Spleen cells, number of mononuclear cells 1.3 × 10^8^ per gram spleen, spleen mass 200 g.^21^ sIL‐6R, values of *Kd* and *B*_max_ from the literature.^4^, ^7^, ^20^

Abbreviations: IL‐6, interleukin‐6; sIL‐6R, soluble IL‐6 receptor.

^a^ Molecular weight set to 50 kDa. 2000 pM ≈ 100 ng/mL.

Example 4 (Soluble IL‐6 receptors, sIL‐6R. *Kd* ≈ 5 nM = 5000 pM (Table 2)):

The degree of saturation at a plasma IL‐6 concentration of 2.0 pg/mL (≈0.08 pM):

$$\frac{B}{B_{\max}}=\frac{F}{\mathrm{Kd}+F}=\frac{0.08\;\text{pM}}{5000\;\text{pM}+0.08\;\text{pM}}=0.0016\%.$$

Under normal physiological conditions, the plasma concentration of sIL‐6R is 35 ng/mL, and since the molecular weight is approximately 50 kDa the maximum binding capacity of plasma sIL‐6R can be estimated to, *B*_max_ ≈ 35 ng/mL × 1000 mL/L/50 000 g/mol = 700 pM. Thus, a saturation of 0.0016% corresponds to 0.011 pM of IL‐6 bound to sIL‐6R. Compared to an *F* of 0.08 pM, this means that there is approximately seven times more IL‐6 in plasma as free IL‐6 as compared with IL‐6 in complex with sIL‐6R (under normal physiological conditions). Nevertheless, in the bloodstream sIL‐6R seemed to have bound almost the same amount of IL‐6 as the entire MNCs together, and approximately three times more than the entire blood granulocytes together.

Analogously, the IL‐6 binding at different concentrations of free IL‐6 was calculated (Table 3). It appears that, for example, the binding of an average of 7.38 IL‐6 molecules per MNC is estimated when the free IL‐6 plasma concentration is 10 pg/mL. At the same plasma concentration of IL‐6, more than twice as many IL‐6 molecules are bound per hepatocyte and only approx. one IL‐6 molecule per PNG. Under physiological conditions, hepatocytes seem to have bound approximately 100 times more IL‐6 than the entire amount of circulating granulocytes (Table 3).

**Table 3:** The binding of IL‐6 in blood in relation to different concentrations of free, measurable plasma IL‐6

*F*, pM	*B*, pM/number of IL‐6 molecules bound per cell				
	MNC	Granulocytes	Hepatocytes	Spleen	sIL‐6R
0.02 (0.5 pg/mL^a^)	0.004/0.38	0.001/0.064	0.09/0.80	0.005/0.38	0.003
0.08 (2 pg/mL)	0.015/1.51	0.004/0.25	0.37/3.18	0.022/1.51	0.01
0.20 (5 pg/mL)	0.037/3.74	0.010/0.63	0.91/7.89	0.054/3.74	0.03
0.40 (10 pg/mL)	0.074/7.38	0.020/1.25	1.79/15.58	0.106/7.38	0.05
1.00 (25 pg/mL)	0.177/17.75	0.048/3.00	4.31/37.50	0.256/17.75	0.13
25.00 (625 pg/mL)	1.769/177.50	0.481/30.00	43.13/375.00	2.563/177.50	3.23

Abbreviations: IL‐6, interleukin‐6; MNC, mononuclear cell; sIL‐6R, soluble IL‐6 receptor.

^a^ The molecular weight of native IL‐6 was set to 25 000 grams per mole.

**Figure 2:**
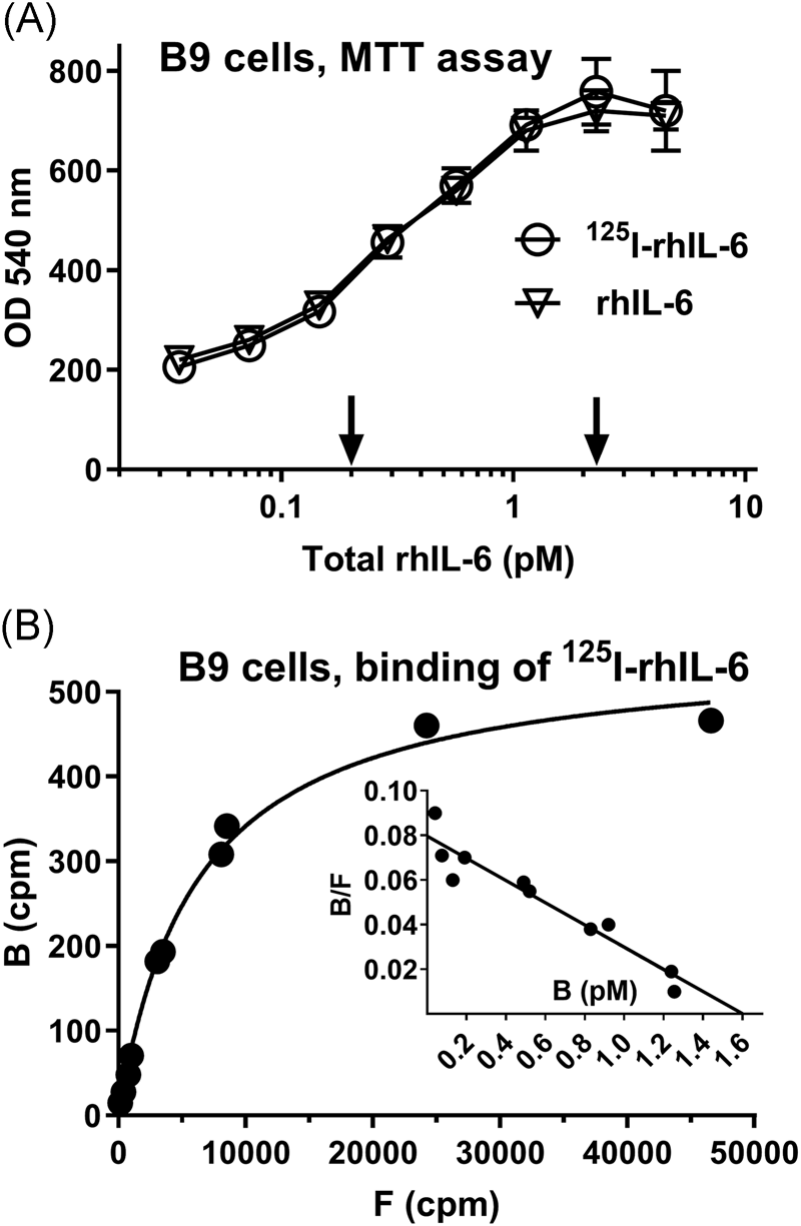
A, ^125^I‐rhIL‐6 was fully bioactive on B9 cells in the MTT assay. Five thousand cells in 110 µL of media supplemented with varying concentrations of labeled and unlabeled rhIL‐6 were incubated for 72 hours at 37°C, after which cellular dehydrogenase activity was assessed via the MTT assay. The two arrows indicate total rhIL‐6 concentrations at which cellular dehydrogenase activity differed significantly (*P* < .01) from that of unstimulated cells (background values), corresponding to 0.2 pM, and the lowest total rhIL‐6 concentration giving rise to maximal dehydrogenase activity, corresponding to 2.3 pM. Data represent the means and SDs of triplicate experiments. The depicted data are representative of four separate experiments with similar outcomes. B, ^125^I‐rhIL‐6 binding to B9 cells. A total of 1.32 × 10^6^ B9 cells in 225 µL of media were incubated with varying concentrations of ^125^I‐rhIL‐6 for 22 hours at 4°C, after which cell‐bound and free ^125^I‐rhIL‐6 were measured. Inset: B9 cells bound ^125^I‐rhIL‐6 with a *Kd* ≈ 20 pM and a *B*_max_ ≈ 1.6 pM, corresponding to 164 binding sites per cell (*R* ^2^ = .92; *P* < .001). The experiment was conducted individually and was repeated four times with comparable results (*Kd* ≈ 17‐23 pM, the number of binding sites per cell ≈ 132‐218). MTT, 3‐(4,5‐dimethylthiazol‐2‐yl)‐2,5‐diphenyltetrazolium bromide; rhIL‐6, recombinant human interleukin‐6

The biological significance of cellular IL‐6 binding was further explored using the IL‐6 sensitive B9 hybridoma cell line and sterile, fully bioactive ^125^I‐labeled rhIL‐6 (Figure 2A). B9 cells require IL‐6 for proliferation and survival, which can be measured by cell viability assays such as the MTT assay.^18^, ^24^ The lowest total IL‐6 concentration (*B* + *F*) at which bioactivity was measurable was 0.2 pM (*P* < .01). The lowest total IL‐6 concentration giving rise to maximal bioactivity was 2.3 pM (Figure 2A). B9 cells displayed on average 164 (*B*_max_) high‐affinity (*Kd* ≈ 20 pM) binding sites per cell (Figure 2B).

**Figure 3:**
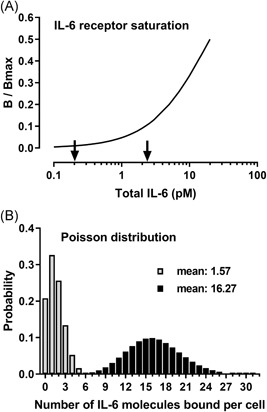
A, Cellular IL‐6 receptor saturation, *B*/*B*_max_, as a function of total IL‐6. Calculations were performed using the data from Figure 2 and the formula described in Section 2. The two arrows indicate total rhIL‐6 concentrations at which cellular dehydrogenase activity was statistically significantly higher (*P* < .01) compared with that of unstimulated cells (0.2 pM) and the lowest total rhIL‐6 concentration that gave rise to maximal dehydrogenase activity (2.3 pM). B, The Poisson distributions for means 1.57 and 16.27. Five thousand B9 cells in 110 µL of media, with each cell having on average 164 binding sites for IL‐6, give rise to a *B*_max_ of 0.012 pM. The binding affinity, *Kd*, was set at 20 pM. A significant bioresponse was observed at an IL‐6 concentration of 0.2 pM. The cellular IL‐6 binding at this concentration was calculated to be 12 × 10^−5^ pM, corresponding to 7867 IL‐6 molecules bound in total or an average of 1.57 IL‐6 molecules bound per cell. The same calculation performed at an IL‐6 concentration of 2.3 pM resulted in a cellular IL‐6 binding of 124 × 10^−5^ pM, corresponding to 81 385 IL‐6 molecules bound in total or an average of 16.27 IL‐6 molecules bound per cell. The Poisson distributions for means 1.57 and 16.27 are shown, which equal the probability (or the proportion) of cells that have bound the indicated number of IL‐6 molecules. rhIL‐6, recombinant human interleukin‐6

Figure 3A shows the degree of saturation of B9 cellular binding sites (*B/B*_max_) as a function of total added IL‐6 (*B* + *F*). At a total IL‐6 concentration of 0.2 pM (corresponding to the cellular IL‐6 sensitivity), the average cellular IL‐6 binding was calculated to be 1.57 (range, 1.08‐2.26) molecules per cell. Accordingly, measurable bioactivity occurred when only 1% (range, 0.82%‐1.04%) of the binding sites were occupied (1.57 IL‐6 molecules/164 binding sites). The lowest total IL‐6 concentration generating maximal bioactivity was 2.3 pM, corresponding to 16.27 (range, 11.47‐23.65) molecules bound per cell on average.

Figure 3B reveals the Poisson distribution for means of 1.57 and 16.27. It appears that 46.5% of the cells bound two or more IL‐6 molecules, and 7.5% of the cells bound four or more IL‐6 molecules at a total IL‐6 concentration of 0.2 pM (corresponding to the cellular IL‐6 sensitivity). Likewise, 99.99% of the cells bound four or more IL‐6 molecules, and 56.0% of the cells bound 16 or more IL‐6 molecules at a total IL‐6 concentration of 2.3 pM. Considering the bioactive cellular IL‐6 receptor system as a hexameric system binding two IL‐6 molecules, the data support the notion that activation of only two functional IL‐6 receptors (corresponding to cellular binding of four IL‐6 molecules) was sufficient to cause measurable bioactivity, whereas activation of only eight IL‐6 receptors appeared to trigger substantial bioactivity in vitro.

## 4 DISCUSSION

This study investigated the potential biological and physiological significance of different concentrations of plasma IL‐6. The nature of this problem is complex and methodologically a reductionist approach was chosen based on simple and logical‐mathematical concepts supplemented with concrete calculations and strengthened by straightforward in vitro IL‐6 binding studies linked to measurements of IL‐6 bioactivity. The methodological approach is in principle applicable to other cytokines. Plasma IL‐6 was chosen since this cytokine is one of the most studied and clinically monitored cytokines, and because the tools for the present investigations such as bioactive iodinated recombinant IL‐6, cell binding assays, and bioassays have been thoroughly validated,^16^, ^20^, ^25^ and since the bloodstream contains IL‐6 receptor‐bearing cells and well‐characterized soluble binding factors.^7^

Together, the data support the notion that the very low concentrations of plasma IL‐6 measured under physiological conditions reflect an endocrine IL‐6 activity on target cells with more than 100 bioactive IL‐6 receptors (corresponding to 200 high‐affinity binding sites per cell), such as hepatocytes and activated T cells, and not least IL‐6 dependent malignant cells, for example, myeloma cells.^26^

The complex nature of the problem required some critical considerations and obviously the experimental approach has some limits. First, the analyses revealed estimates, based on the assumptions that available cellular receptors were evenly distributed on the target cells and that the binding equilibria in plasma were completed, and this will, per definition, never be the case. However, based on published data based on flow cytometry^11^ and the rapidly occurring association kinetics,^15^, ^16^ it is a rational assumption that the estimates described here is representative for more than 90% of the binding phenomena. Second, the binding experiments shown here were conducted at 4°C, whereas bioactivity was assessed at 37°C. Specific cell binding of IL‐6 in terms of affinity and maximal binding capacity is temperature independent.^27^ The affinity (*Kd*) is related to free energy difference (Δ*G* ^0^) between the initial and the final state of the binding reactions, whereas only the association‐ and dissociation‐rates are related to the temperature. B9 cell IL‐6 binding equilibrium data were also tested at 22°C and 37°C, and no significant differences in *Kd* and *B*_max_ were observed (data not shown). This is in line with other observations^27^ and the observed cellular IL‐6 receptor turnover of several hours.^6^, ^16^, ^28^, ^29^ Hence, the overall estimates and conclusions seem reasonable.

Microscopic examinations of IL‐6‐dose‐MTT‐responses at intermediate IL‐6 concentrations (Figure 2A) revealed a mixed scenario with some of the cells showing full metabolic activity and with less or no metabolic activity in the remainder of the cells (data not shown) underpinning the stochastic nature of IL‐6 stimulation under the given experimental conditions. To more precisely determine the heterogeneity in cellular responses to IL‐6, however, single‐cell experiments are required, such as flow cytometry with proliferation and survival markers.

IL‐6 also signals intracellularly from within endosomes^30^ and about half of the cellular IL‐6 receptors might reside in intracellular endosomes where IL‐6 signaling also leads to degradation of the receptor. Obviously, the basic equilibrium principles lined out in this paper also apply to this kind of intracellular cytokine signaling. However, the degree of intracellular signaling does not affect the overall interpretations from this study which focused exclusively on extracellular free and measurable cytokines in the context of extracellularly available cytokine receptors and binding factors.

The Poisson distribution was used to statistically qualify what an average of 1.57 or 16.27 IL‐6 molecules bound per cell implied, based on the following assumptions: (a) the number of occupied IL‐6 binding sites could be from 0, 1, 2, … 164; (b) cellular IL‐6 binding occurred independently to the available binding sites; (c) the cellular binding sites were equally distributed among the cells; and (d) only two IL‐6 molecules could bind to one and the same high‐affinity receptor system. This analysis revealed that stimulating only two functional receptors was sufficient to elicit a measurable biological response in vitro. Under the given experimental conditions and at a total IL‐6 concentration as low as 0.2 pM (corresponding to 4.5 pg/mL), a 15% to 20% increase in bioactivity was observed (Figure 2A). At this IL‐6 concentration, approximately 7.5% of the cells bound four or more IL‐6 molecules. Figure 3A shows, that when approximately 10% of the cellular binding sites were occupied, corresponding to eight functional IL‐6 receptors, bioactivity reached its maximum. These observations verify the well‐known high IL‐6‐sensitivity of B9 cells. The data also demonstrate a nonlinear relationship between receptor occupancy and bioactivity and a large receptor reserve even though the number of cellular IL‐6 receptors was relatively low.

Obviously, receptor binding of 1.57 IL‐6 molecules does not make sense in a stoichiometric and hence biological setting. The figure must be understood in the context of the uncertainties that the measurements and thus the calculations give rise to, just as the number must be matched against the composition of the cellular IL‐6 receptor complex. The number of IL‐6 molecules bound per cell should be a multiple of 2. All binding experiments in this paper was carried out with radio‐iodinated, *E. coli*‐derived rhIL‐6. Even though the labeling of IL‐6 had no measurable impact on the biological activity (Figure 2A) it should be considered that natural IL‐6, in contrast to recombinant IL‐6, is heavily glycosylated. It is therefore plausible that the native glycosylated IL‐6 binds with a higher affinity to cellular receptors.^20^ Taking these considerations into account, the present data indicate that activation of all the way down to a single receptor complex might be sufficient to trigger cellular signaling while activating three receptor complexes per cell (corresponding to the binding of six IL‐6 molecules) with certainty induces a biological signal.

Interestingly the number of IL‐6 binding sites on B9 cells was just in between the number of the binding sites measured on MNC and PNG and in accordance with reported cellular cytokine receptors on leukemia and lymphoma cells. Uckun et al^31^ observed a single class of 132 to 154 high‐affinity IL‐1 receptors per cell on IL‐1‐responsive leukemia cells, and the IL‐1 concentrations required for half‐maximal receptor occupancy were approximately three orders of magnitude higher than those needed to elicit a half‐maximal proliferative response, underscoring that only a small fraction of IL‐1 receptors needed to be occupied to stimulate a substantial bioresponse. In addition, the number of cellular high‐affinity receptors for IL‐3, G‐CSF, and GM‐CSF on various leukemic cell lines varied between 30 and 450.^32^

The 150 to 200 IL‐6 binding sites per B9 cell, corresponding to 75 to 100 functional IL‐6 receptors, is much lower than the number of IL‐6 receptors on hepatocytes and myeloma cells, which express several hundred to thousands of functional IL‐6 receptors.^22^, ^26^, ^33^ By extension, such cells should be extremely IL‐6 sensitive if activation of only two to eight receptors is required to elicit a biological response. In this context the estimated dominant IL‐6 binding in the liver was remarkable. Under physiological conditions, by far the major part of the total body amount of IL‐6 seems to be bound to hepatocytes due to their abundance and their high number of IL‐6 receptors (Table 2). This dominant binding has been confirmed experimentally by in vivo measurements supporting the notion that this organ is a prime IL‐6 target organ producing acute phase proteins and mainly responsible for removing IL‐6 from the circulation.^28^, ^33^

**Figure 4:**
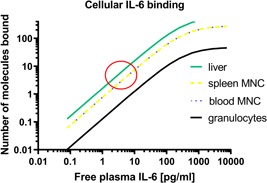
A theoretical calculation of how many IL‐6 molecules are bound to various target cells at different concentrations of free plasma IL‐6. The red circle marks the range of physiological plasma IL‐6 concentrations and the concomitant number of IL‐6 molecules bound per cell. Hepatocytes and MNC are likely IL‐6 stimulated whereas circulating PNG is unstimulated under normal physiological conditions. Circulating PNG is activated only when measurable plasma levels of free IL‐6 rise above 50 pg/mL. IL‐6, interleukin‐6; MNC, mononuclear cell; PNG, polymorph nuclear granulocyte

Normal plasma IL‐6 levels vary very little during the day and are between less than 0.02 pg/ml and approximately 5.0 pg/mL (5.0 fM and 0.2 pM).^20^, ^34^ This means, that MNC, such as circulating T lymphocytes and hepatocytes in direct contact with plasma seems to have captured enough IL‐6 to be bioactivated under normal physiological conditions (Figure 4). This notion was recently supported by the observation that blood donors with high levels of neutralizing autoantibodies against IL‐6 and hence, unmeasurable levels of free plasma IL‐6 had markedly lower levels of plasma C‐reactive protein as compared with antibody‐negative blood donors.^35^

The data and the estimations in this study underscore the well‐established biopotency of cytokines and it puts into perspective the countless studies on experimental and clinical cytokine measurements. It points to the importance of knowing the nature of a measured cytokine, for example, is the cytokine free or is it bound to soluble binding factors? The notion that fundamental cytokine‐mediated biological processes appear to be driven by femtomolar to picomolar extracellular levels of cytokines means that we should critically interpret in vitro generated data based on nanomolar cytokine stimulation, which is thousands to a million times higher than the levels that seem to prevail under physiological and most pathophysiological situations.

## CONFLICT OF INTERESTS

The authors declare that there are no conflict of interests.

## Data Availability

The data that support the findings of this study are available from the corresponding author upon reasonable request.
